# Comparative Assessment of Commercial and Waste-Derived Sodium Silicate Activators in PFA-GGBS Alkali-Activated Concrete

**DOI:** 10.3390/ma19204321

**Published:** 2026-10-14

**Authors:** Andrew Ayres, Jonathan Oti, John Kinuthia

**Affiliations:** Faculty of Computing, Engineering and Science, University of South Wales, Pontypridd CF37 1DL, UK; john.kinuthia@southwales.ac.uk

**Keywords:** sodium silicate (SS), sodium hydroxide (SH) alkali-activator, waste-derived activator, ground granulated blast furnace slag (GGBS), pulverized fuel ash (PFA), silica fume (SF), compressive strength, workability, setting time, alkali-activated binder (AAB), alkali-activated concrete (AAC)

## Abstract

The growing demand for Portland cement has intensified concerns over construction-related CO_2_ emissions, driving interest in low-carbon alternatives such as alkali-activated concrete (AAC). Although AAC can substantially reduce embodied carbon, the production of commercial sodium silicate (SS), a widely used activator, remains a significant environmental burden. This study evaluates the feasibility of a waste-derived sodium silicate activator produced from silica fume (SF) and sodium hydroxide (NaOH) and compares its performance with that of a conventional commercial activator in blended f fuel ash (PFA) and ground granulated blast furnace slag (GGBS) systems. Fresh and hardened properties were assessed across a range of activator-to-binder (A/B) and SS/SH ratios, considering workability, setting time, and compressive strength requirements for structural concrete applications. Increasing the A/B ratio from 0.16 to 0.20 improved compressive strength and accelerated setting, while higher SS/SH ratios enhanced early- and later-age strength through increased soluble silica availability and gel formation. Although higher activator contents reduced workability due to increased viscosity, both commercial and SF-derived activators achieved comparable mechanical performance. The high amorphous silica content of SF was found to promote dense C-(A)-S-H gel development, while 10 M NaOH and a 24 h ambient-temperature extraction process provided sufficient silica dissolution without additional heating. These findings demonstrate the potential of SF-derived sodium silicate as a sustainable activator for producing low-carbon AAC with performance comparable to conventional systems.

## 1. Introduction

The construction industry is under increasing pressure to reduce greenhouse gas emissions and improve resource efficiency. Portland cement (PC), the primary binder used in conventional concrete, is responsible for approximately 7–8% of global anthropogenic CO_2_ emissions due to the energy-intensive calcination of limestone and clay and the associated combustion of fossil fuels during manufacture [1]. Although the incorporation of supplementary cementitious materials such as pulverized fuel ash (PFA), ground granulated blast furnace slag (GGBS), and silica fume (SF) has reduced clinker consumption, the development of alternative low-carbon binders remains a critical research priority [2,3].

Among the emerging alternatives, alkali-activated concrete (AAC) has attracted significant attention owing to its ability to utilize industrial by-products and waste materials while achieving substantial reductions in embodied carbon compared with Portland cement concrete [4,5]. Previous studies have demonstrated that AAC can provide favorable engineering properties, including high compressive strength, rapid strength development, and enhanced durability [6,7,8,9,10,11,12]. However, despite these advantages, the environmental benefits of AAC are often compromised by the alkali activator.

It has been widely reported that alkali activators can contribute a significant proportion of the total embodied carbon of AAC mixtures, accounting for up to 75% in some applications [13,14]. Sodium silicate, one of the most effective and widely used activators, is generally associated with the highest environmental burden due to its energy-intensive manufacturing process, which requires temperatures exceeding 1500 °C and results in substantial CO_2_ emissions [14]. This apparent contradiction has led to growing interest in developing more sustainable and economically viable activators derived from industrial and agricultural waste streams [15,16]. Among these alternatives, silica fume is particularly attractive because of its high amorphous silica content and widespread availability as an industrial by-product.

Despite increasing research into waste-derived activators, studies directly comparing the engineering performance of silica fume-derived sodium silicate with conventional commercial activators remain limited [16,17,18,19,20,21]. Furthermore, although previous studies have demonstrated the feasibility of producing sodium silicate activators from silica fume and successfully applying them in alkali-activated materials [15,22,23,24], existing research has primarily focused on activator synthesis, proof-of-concept validation, mixing methodologies, and precursor optimization. As a result, limited guidance is currently available regarding the optimization of activator composition for practical concrete applications.

To further clarify the development of this research area, Table 1 summarises the objectives and contributions of previous studies together with the scope of the present investigation. Whilst earlier studies successfully demonstrated the technical feasibility of silica fume-derived sodium silicate activators, important knowledge gaps remain regarding their optimization and practical implementation within structural alkali-activated concrete systems. Direct benchmarking against commercially available sodium silicate activators has received limited attention, leaving uncertainty regarding the suitability of waste-derived activators as a replacement for conventional activator systems. Consequently, further research is required to establish practical activator dosage ranges and determine whether comparable fresh and hardened concrete performance can be achieved using a silica fume-derived alternative.

Therefore, the novelty of the present study lies not in the synthesis of the silica fume-derived sodium silicate activator itself, but in the systematic evaluation of the activator performance within a PFA-GGBS alkali-activated concrete system. Specifically, this study investigates the combined influence of the sodium silicate-to-sodium hydroxide ratio (SS/SH) and activator-to-binder ratio (A/B) on workability, setting behaviour, and compressive strength, whilst benchmarking the performance of a waste-derived sodium silicate activator against a commercially available sodium silicate activator. By establishing practical activator dosage ranges and identifying performance relationships between activator composition and engineering properties, the study provides implementation-focused guidance for future mix design and application of waste-derived activators in alkali-activated concrete.

## 2. Materials and Methods

### 2.1. Materials

**Figure 1:**
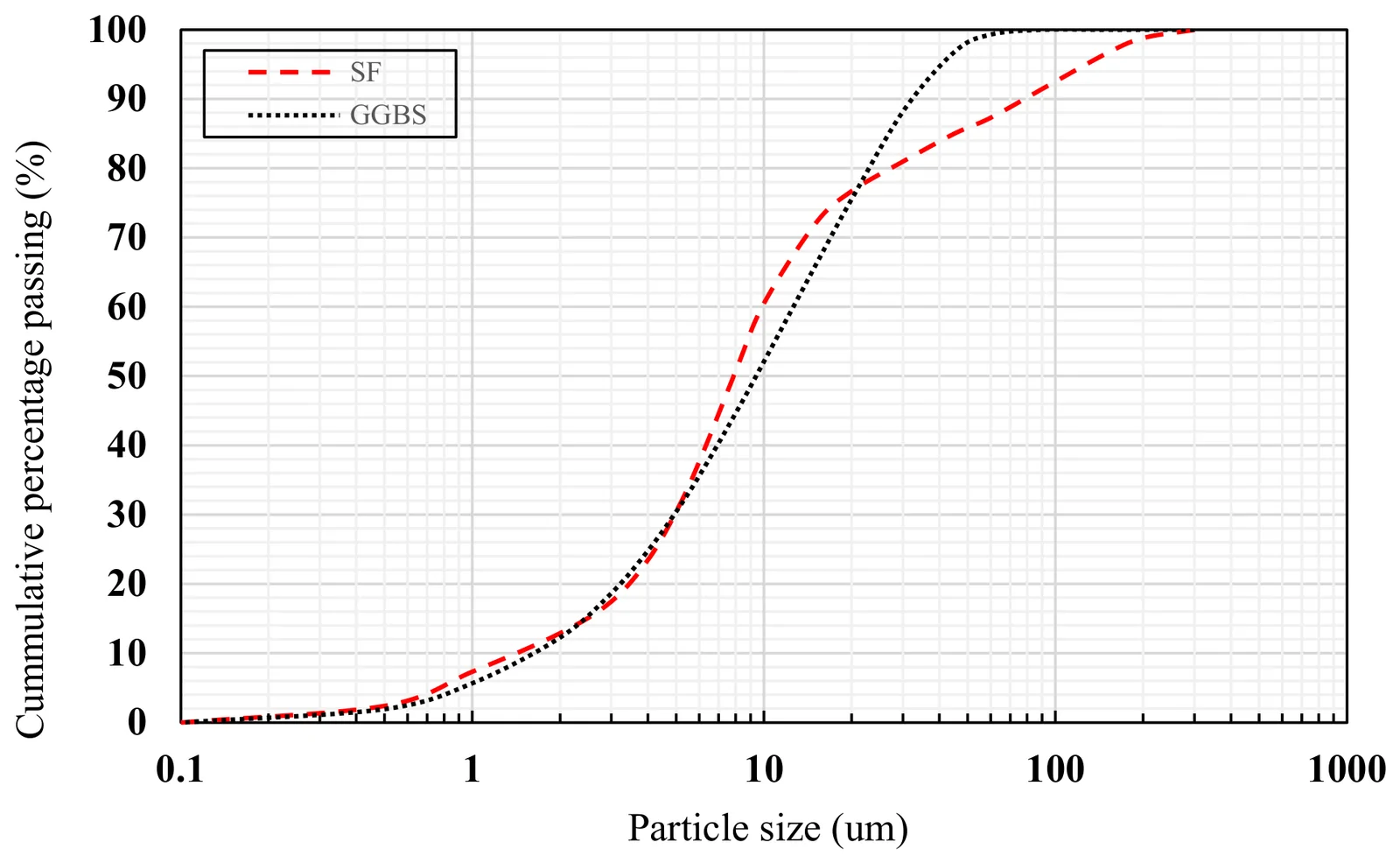
Particle size distribution curves for binder materials.

The current study utilized pulverized fuel ash (PFA), ground granulated blast furnace slag (GGBS), silica fume (SF), sodium hydroxide (NaOH), sodium silicate (SS), and aggregates. All material properties presented in this study were obtained from the respective suppliers and manufacturers. PFA was supplied by CEMEX, Cardiff, UK, and manufactured in accordance with BS EN 450-1:2012 [25]. The material was light grey in colour and comprised principally of SiO_2_ (59.04%) and Al_2_O_3_ (34.08%). Ground granulated blast furnace slag (GGBS) was supplied by Tarmac Cement Division, Birmingham, UK, in accordance with BS EN 15167-1:2006 [26]. The material was off-white in colour and was characterized by a D_50_ particle size of 9.40 μm. Silica fume was supplied by Tarmac Cement and Lime Company, Derbyshire, UK, under the trade name Elkem MICROSILICA 971U and manufactured by Elkem Silicon Materials, Kristiansand, Norway. The material complied with BS EN 13263-2:2005 + A1:2009 [27] and possessed a SiO_2_ content of approximately 97.1%. The oxide compositions of the precursor materials are presented in Table 2, while their physical properties and particle size distributions (PSD) are shown in Table 3 and Figure 1, all of which were provided by the suppliers referenced in this study.

**Table 2:** Oxide composition of binders.

Oxide	Composition (wt %)		
	PFA ^(a)^	GGBS ^(b)^	SF ^(c)^
CaO	0.22	37.99	0.29
SiO_2_	59.04	35.54	97.1
Al_2_O_3_	34.08	11.46	0.23
MgO	-	8.78	0.22
Fe_2_O_3_	2.00	0.42	0.07
Mn_2_O_3_	-	0.43	-
SO_3_	0.05	1.54	0.18
TiO_2_	-	0.70	-
K_2_O	3.02	0.43	0.35
Na_2_O	1.26	0.37	0.25

Data provided by the suppliers; ^(a)^ CEMEX, Cardiff, ^(b)^ UK Tarmac Cement Division, Birmingham, UK, ^(c)^ Elkem Silicon Materials, Norway.

**Table 3:** Properties of binders.

Properties	PFA ^(a)^	GGBS ^(b)^	SF ^(c)^
Grain size range (µm)	-	0.5–300	0.1–300
D10	-	1.65	1.6
D50	-	9.40	8.2
D90	-	32.04	82.6
Color	Light Grey	Off-white	Light Grey
Particle density (kg/m^3^)	2300	2970	2370

Data provided by the suppliers: ^(a)^ CEMEX, Cardiff, ^(b)^ UK Tarmac Cement Division, Birmingham, UK, ^(c)^ Elkem Silicon Materials, Norway.

**Figure 2:**
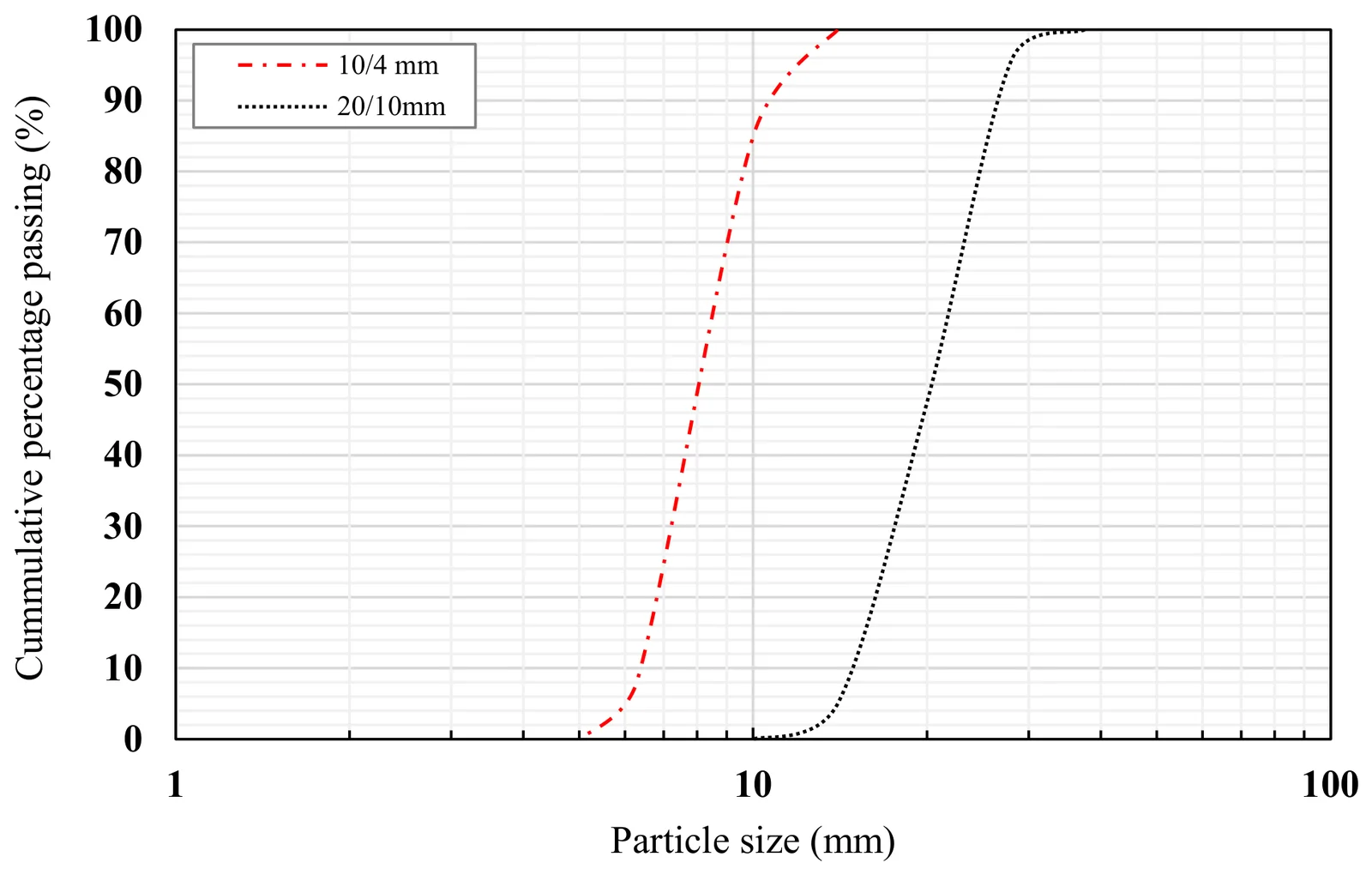
Particle size distribution curves for coarse and fine aggregates.

Crushed limestone aggregates of two size fractions (20/10 mm and 10/4 mm) were employed as coarse aggregate in both the ordinary Portland cement (OPC) control mixtures and the alkali-activated concrete (AAC) formulations. The aggregates were supplied by Jewson Ltd., South Wales, UK, and produced in accordance with BS EN 12620:2013 [28]. Figure 2 presents the particle size distribution curves of the aggregates, while their physical and mechanical properties are summarised in Table 4.

The alkaline activator system consisted of commercially available sodium silicate solution and sodium hydroxide. The sodium silicate solution had a SiO_2_:Na_2_O ratio ranging between 1.5 and 2.5 and comprised 25.5% SiO_2_, 12.8% Na_2_O, and 61.7% H_2_O, with a density of 1560 kg/cm^3^. Sodium hydroxide was supplied in pellet form with a purity of 99% and was dissolved in deionized water to produce a 10 M solution. Both products were obtained from Fisher Scientific Ltd., Loughborough, UK.

An alternative sodium silicate solution was synthesized using silica fume and 10 M sodium hydroxide solution. The activator was prepared by dissolving undensified silica fume (971U) in sodium hydroxide solution to produce a sodium silicate-like solution with a SiO_2_:Na_2_O molar ratio of approximately two. The density of the synthesized solution was comparable to that of the commercial sodium silicate solution and was subsequently used as an alternative activator in the alkali-activated mixtures (refer to Table 5).

### 2.2. Methodology

#### 2.2.1. Mix Design

The basic principle that underpins this procedure is orientated towards achieving specific minimum performance-based criteria (≥C32/40 compressive strength class under BS 8500:2019 [29], S4 consistency slump class BS EN 12350-2 [30] and a minimum initial setting time ≥45 min in accordance with PAS 8820:2016 [31]), with the aim to conduct future pilot trials as part of the HS2 Innovation Programme.

The alkali activator dosage employed is defined in two parts: (i) sodium silicate and sodium hydroxide ratio (SS/SH) and (ii) total alkali activator to binder ratio, as this approach is widely used by the research community, noting that these are the driving parameters for microstructural and mechanical development in alkali-activated systems. The water-to-binder ratios employed measure the water consumed against the total binder content. This method is traditionally used for OPC technology and has been widely used for AAC technology [15,17]. In terms of mix composition, all specimens investigated have a fixed 1:2:3 binder–sand–aggregate for concrete and 1:2.5 binder–sand for mortar relating to setting time. Table 6 summarises the two activator regimes investigated in this study. Regime A utilized a conventional commercial sodium silicate activator, whereas Regime B utilized a silica fume-derived sodium silicate activator prepared in the laboratory. All mix constituents are defined in Table 7 through the reported material quantities, activator-to-binder ratio, and sodium silicate-to-sodium hydroxide ratio, which together fully specify the mixture compositions.

**Table 6:** The activator regimes used in this study.

Mix Regime	Details
Regime A	Commercial sodium silicate and sodium hydroxide solution
Regime B	(Silica Fume + NaOH) and sodium hydroxide solution

**Table 7:** AAC mix design for 1 m^3^ with respect to SS/SH ratios 3:1, 2.25:1, and 1.50:1 and the ratio (AB) 0.16, 0.18, and 0.20.

Mix Code	Alkali-Activated Binder				Water (kg/m^3^)	Aggregates (kg/m^3^)		
	GGBS (kg/m^3^)	PFA (kg/m^3^)	A/B Ratio	SS:SH Ratio		Sand	10 mm	20 mm
AA.01	225.4	150.3	0.16	3.00	149.5	751.2	450.72	676.08
AA.02	225.4	150.3	0.16	2.25	149.5	751.2	450.72	676.08
AA.03	225.4	150.3	0.16	1.50	149.5	751.2	450.72	676.08
BB.01	225.4	150.3	0.18	3.00	149.5	751.2	450.72	676.08
BB.02	225.4	150.3	0.18	2.25	149.5	751.2	450.72	676.08
BB.03	225.4	150.3	0.18	1.50	149.5	751.2	450.72	676.08
CC.01	225.4	150.3	0.20	3.00	149.5	751.2	450.72	676.08
CC.02	225.4	150.3	0.20	2.25	149.5	751.2	450.72	676.08
CC.03	225.4	150.3	0.20	1.50	149.5	751.2	450.72	676.08

#### 2.2.2. Preliminary Trials

To validate the fresh and hardened properties of AAC chemically activated using Regime B, four preliminary trial mixtures were selected for the initial investigation. In doing so, a 40% PFA: 60% GGBS (% of the total binder wt.) binder composition was selected based on earlier recommendations given by [32] for achieving satisfactory material handling time. Secondly, a suitable activator dosage that includes an SS/SH ratio = 2:5 was expected to provide exceptional material performance, as this has been suggested by [33] to be the optimal ratio for mechanical performance. Furthermore, recommendations on a suitable activator/binder ratio in the literature vary, depending on the binder composition [34]. Therefore, the A/B ratios selected for initial investigation were 0.20 and 0.25 with the aim of minimizing the overall cost and carbon emissions based on satisfactory results given by [21]. For workability understanding purposes, the opening trials further tested water/binder ratios of 0.40 and 0.42, given that such parameters have been recommended in the literature to achieve high flow [12,22].

#### 2.2.3. Mix Proportion for Assessing the Effects of Activator Dosage on Alkali-Activated Concrete and Mortar Properties (Regime B)

Assessment of the fresh and hardened properties, with emphasis on the sodium silicate and sodium hydroxide ratio (SS/SH) 3:1, 2.25:1, and 1.50:1 in relation to activator/binder ratio (AB) 0.16, 0.18, and 0.20, was analyzed (see Table 8). The precursor selected for this review used a 40:60 PFA: GGBS blend proportion using a fixed *w*/*b* ratio of 0.40 based on initial lab trials, providing satisfactory workability and material handling time. This investigation includes compressive strength development (moisture curing period of 3, 7, and 28 days) and workability (recorded at 5 min) of AAC and initial/final setting time of AAM for validation purposes.

#### 2.2.4. Mix Proportion for Comparison Analysis of Alkali-Activated Concrete and Mortar Properties Using Regime A and B

The optimal activator dosage selected for control purposes was previously nominated (A/B ratio = 0.18 and SS/SH ratio = 2.25) combined with a 40:60 PFA: GGBS mix blend proportion and w/b ratio 0.40. However, for comparative purposes between the two systems (see Table 8), this section focuses on the evaluation and comparison of SS:SH ratios of 3.00, 2.25, and 1.50.

### 2.3. Preparation of Alkali Activators

The sodium hydroxide solution was prepared by dissolving 400 g of NaOH pellets (99% purity) in 1 liter of deionized water to obtain the desired 10 M concentration, based on the recommendations of [35]. The sodium silicate solution used in this study was prepared by Fisher Scientific, Loughborough, UK, consisting of a SiO_2_/Na_2_O molar ratio ranging from 1.5 to 2.5.

(1) NaOH(s) + H_2_O(l) → NaOH(l)

An alternative sodium silicate solution (SSA) was developed for the SF-NaOH gel to replace commercial sodium silicate. The gel was prepared according to Equation (2) to achieve an approximate molar ratio (SiO_2_/Na_2_O) of 2.00 (x = 2).

(2) 2SiO_2_(s) + 2NaOH(l) → Na_2_O(SiO_2_)2(l) + H_2_O(l)

Both SH and SF-NaOH gel were stored in a sealable container for 24 h at room temperature (20 ± 2 °C). SH produces an exothermic reaction when mixed in water, whereby the solution is required to cool down to room temperature to accomplish chemical equilibrium [15]. It is worth mentioning that the initial density of the SF-NaOH solution, after reaching the necessary temperature, required adjustment with additional deionized water for dilution purposes to that of the commercially available SS solution (1560 kg/m^3^). The SH density was also recorded at 1330 kg/m^3^, and all density parameters were calculated according to Equation (2).

### 2.4. Preparation of Mortar Specimens and Testing Methods

Mortar specimens were prepared to assess fresh properties, with emphasis on initial and final setting times. Mixing was performed using a Hobart HSM40-F1E mixer (40 L capacity) at 68 rpm, supplied by Hobart UK, Barnstaple, UK. Binder materials were dry-mixed for 1 min, followed by the addition of sand and mixing for a further 1 min. The activating solution and additional water were then added and mixed for 2 min, after which the mixture was scraped and mixed for a further 2 min to ensure homogeneity. Immediately after mixing, the fresh alkali-activated mortar was placed into a lightly greased 80 mm diameter mold, filled slightly above the rim, and leveled using a steel spatula in accordance with BS EN 480 2:2006 [36]. Following homogenization, the initial setting time of the fresh geopolymer mortar was determined using an automatic Vicat penetrometer (Vicatronic ELE, Leighton Buzzard, Bedfordshire, UK) at an ambient temperature of 23 ± 2 °C in accordance with BS EN 196 3:2016 [37].

### 2.5. Preparation of Concrete Specimens and Testing Methods

The alkali-activated concrete was prepared using a controlled mixing regime. Dry constituents (cement, precursor, fine and coarse aggregates) were accurately weighed according to the proportions listed in Table 7, and mixed using a Croker Cumflow RP100 XD MK2 mixer (operating at 16 rpm), sourced from Winget Limited, located at Bolton, Lancashire, UK.

To determine the compressive strength of hardened concrete, the material was cast into PVC cube molds (100 × 100 × 100 mm), with three samples per mixture taken and compacted in three layers under vibration for 2 min. Specimens were sealed with plastic film to prevent moisture loss and stored at 20 ± 2 °C for 24 h. After 48 h, each specimen was demolded and subsequently cured in sealed containers at 20 ± 2 °C to maintain 90% relative humidity.

The hardened properties of all concrete specimens were evaluated in terms of compressive strength in accordance with BS EN 12390-3:2019 [38] at curing ages of 3, 7, 14, and 28 days. For each mix composition and curing age, three cube specimens were tested, and the reported values represent the mean compressive strength. The figures presented in the study show mean values obtained from repeated tests. Error bars, standard deviations, and confidence intervals were not included because individual specimen measurements were not recorded separately in a form that permitted reliable statistical analysis. Consequently, the mean values are used to highlight comparative performance trends among the investigated mixes rather than to quantify experimental variability.

The workability of the fresh concrete was assessed using the slump test in accordance with BS EN 12350 2:2019 [30]. Fresh concrete was placed in a standard slump cone in three successive layers; each layer being compacted before the cone was carefully lifted vertically. The slump value, measured as the vertical reduction in height of the concrete, was used as an indicator of consistency and workability.

## 3. Results & Discussion

### 3.1. Effects of Activator Dosage on Alkali-Activated Concrete and Mortar Properties (Regime B)

Assessment of the fresh and hardened properties, with emphasis on the sodium silicate and sodium hydroxide ratio (SS/SH) 3:1, 2.25:1, and 1.50:1 in relation to activator/binder ratio (AB) 0.16, 0.18, and 0.20, was analyzed. The precursor selected for this review used a 40:60 PFA: GGBS blend proportion using a fixed w/b ratio of 0.40 based on initial lab trials, providing satisfactory workability and material handling time. This investigation includes compressive strength development (moisture curing period of 3, 7, and 28 days) and workability (recorded at 5 min) of AAC and initial/final setting time of AAM for validation purposes.

#### 3.1.1. Compressive Strength

**Figure 3:**
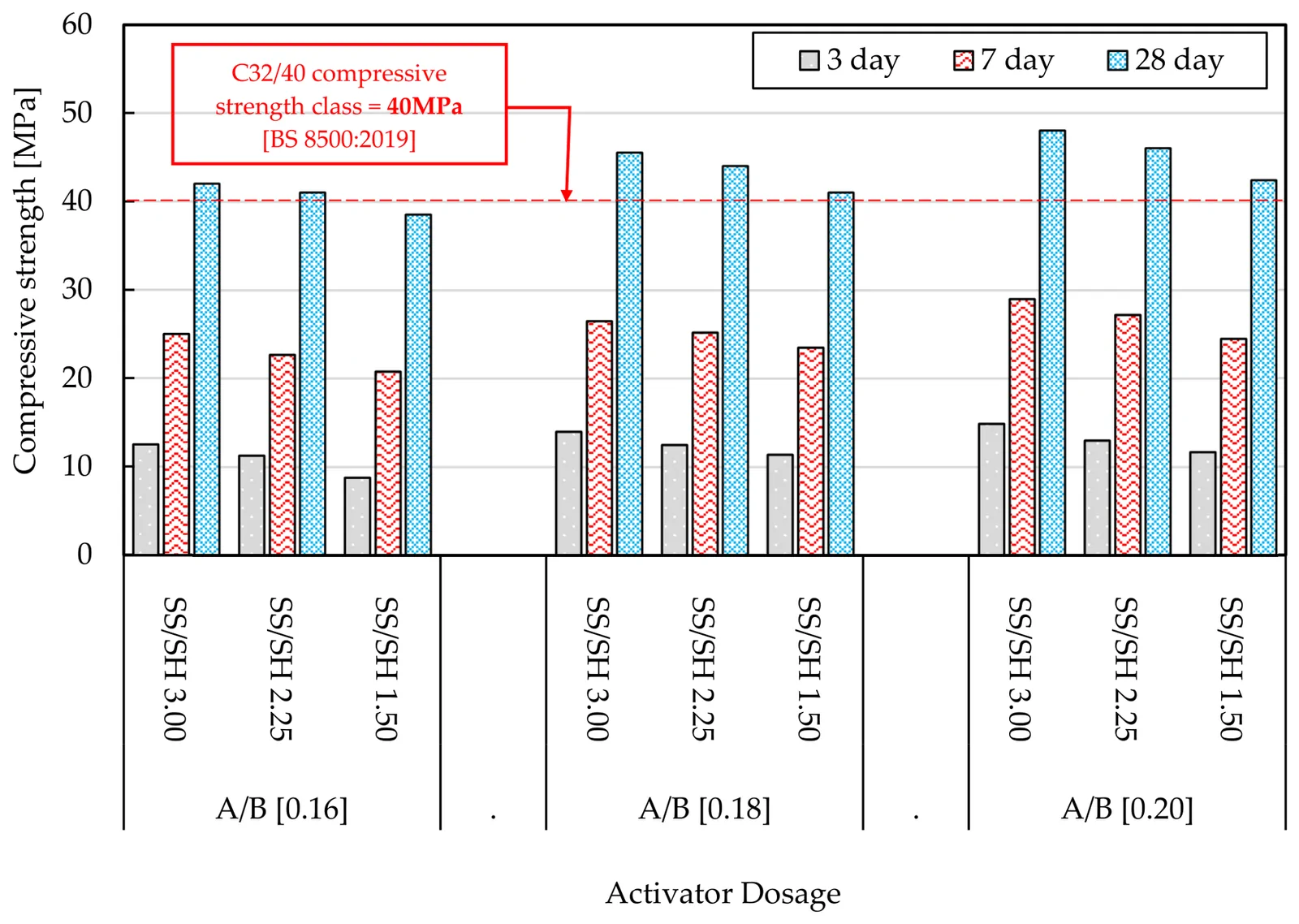
Effect of sodium silicate and sodium hydroxide ratio (SS/SH) for each activator/binder ratio (AB) on compressive strength development for Regime B using a 40:60 PFA: GGBS blended AAC and w/b ratio 0.40.

A direct enhancement in 3-, 7-, and 28-day CS was observed in Figure 3, corresponding to incremental increases in the A/B ratio from 0.16 to 0.18 and 0.20. This could be attributed to the hydration reactions, whereby increasing the A/B ratio naturally enhances the Na+ ion concentration that subsequently increases the pH level, which in turn accelerates the dissolution of silica, alumina, and calcium from the binder system [39]. The enhanced dissolution later promotes the formation of a denser homogeneous matrix structure that leads to higher compressive strength [33,40]. Increasing the SS/SH ratio from 1.5 to 3.0 consistently enhanced strength development across all curing ages. This can be attributed to the increased presence of soluble silica content available from the existence of the larger SS/SH ratio, which in turn can improve the polymerization process by strengthening the Si-O-Si bonds and promoting the formation of a homogeneous structure, leading to higher mechanical strength [41]. This is directly in line with a wide range of authors [23,42,43] stating that increasing the SS/SH ratio develops higher compressive strength for a wide range of AAC compositions. However, it is worth mentioning that [33] stated this will depend on the mix composition’s physical and mineralogical properties. Ref. [33] went on to propose that an SS/SH ratio ≥ 2.5 can, in some cases, have either a negative or neutral effect on the compressive strength.

#### 3.1.2. Workability

**Figure 4:**
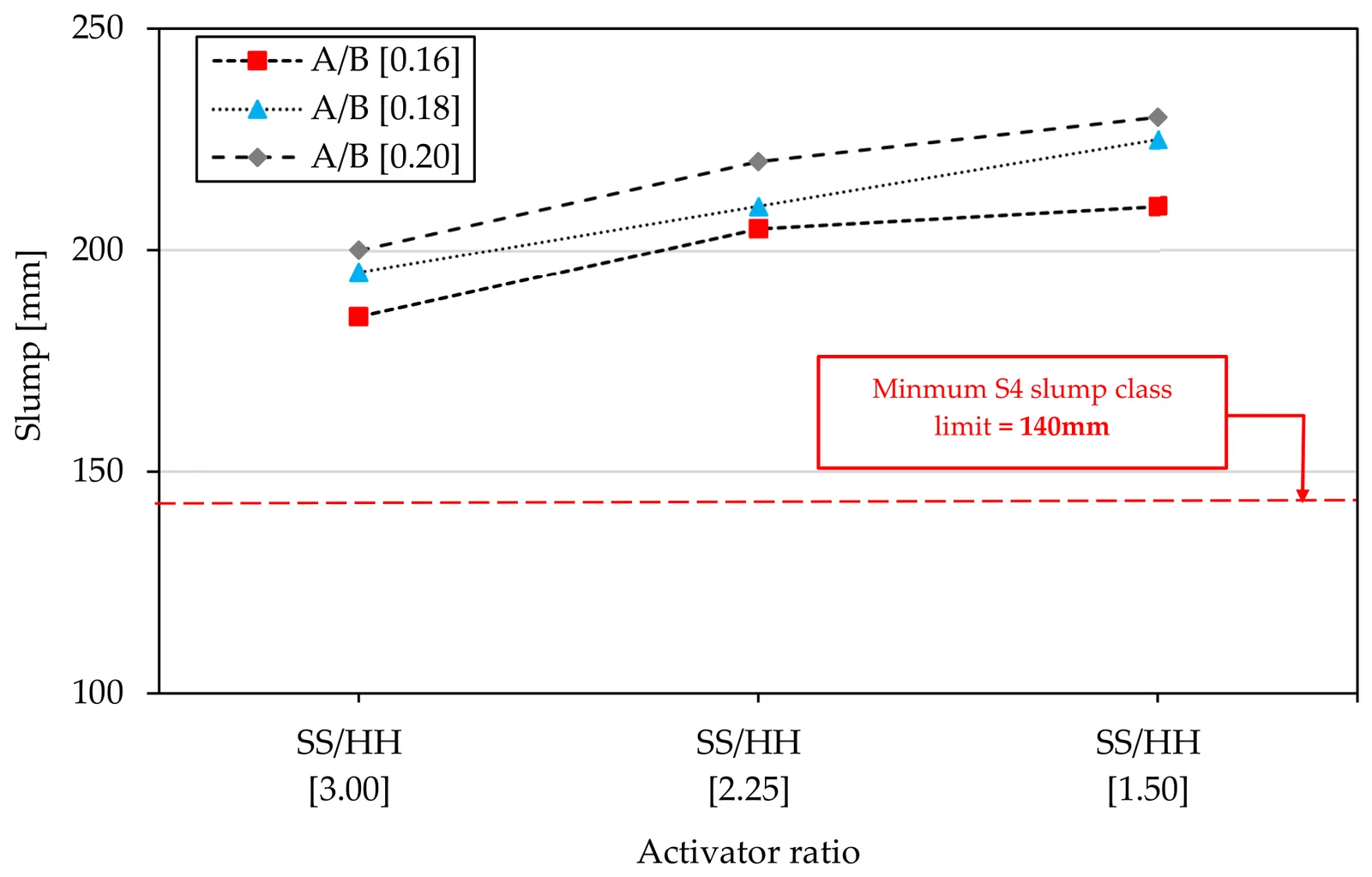
Effect of sodium silicate and sodium hydroxide ratio (SS/SH) for each activator/binder ratio (AB) on workability for Regime B using a 40:60 PFA: GGBS blended AAC and w/b ratio 0.40.

Figure 4 presents the workability (slump) results for AAC mixes produced using the same mix design parameters. All mixes satisfied the minimum S4 slump class requirement of 140 mm in accordance with BS EN 12350-2 [30]. With respect to the reduction in slump values corresponding with the ever-increasing SS:SH ratio from 1.50 to 3.00, this observation could be attributed to the larger SS content present, which in nature is higher in viscosity compared with sodium hydroxide, which would lead to a stickier material, thereby reducing the overall slump consistency [44]. Another theory that might support this argument could be attributed to the rate of reaction caused by an increased presence of soluble silica, which subsequently increases the fresh properties’ viscosity and reduces the workability [45]. This directly corresponds with several researchers [16,46,47], all demonstrating a reduction in slump subject to a higher SS/SH ranging from 1 to 3.5. Another important point to be raised on the earlier observations relevant to the slump was the apparent increase in slump when enlarging the A/B ratio from 0.16 to 0.18 and 0.20. This observation could be attributed to the reduction in total liquid-to-binder ratio, as this can trigger larger frictional force between the material particles during mixing [48], thus reducing the consistency [49]. This is in agreement with [15], who investigated the fresh and hardened properties of alkali-activated slag concrete using a silica fume-derived sodium silicate and sodium hydroxide 10 M solution, reporting a gradual reduction in workability by reducing the A/B ratio from 0.9 to 0.5. [45] reported on the workability of alkali-activated fly ash/slag using various alkali activator contents of 35%, 40%, and 45% of the total binder weight, which dramatically decreases the workability (slump reduced from 230 to 100 mm) when decreasing the activator solution.

#### 3.1.3. Setting Time

**Figure 5:**
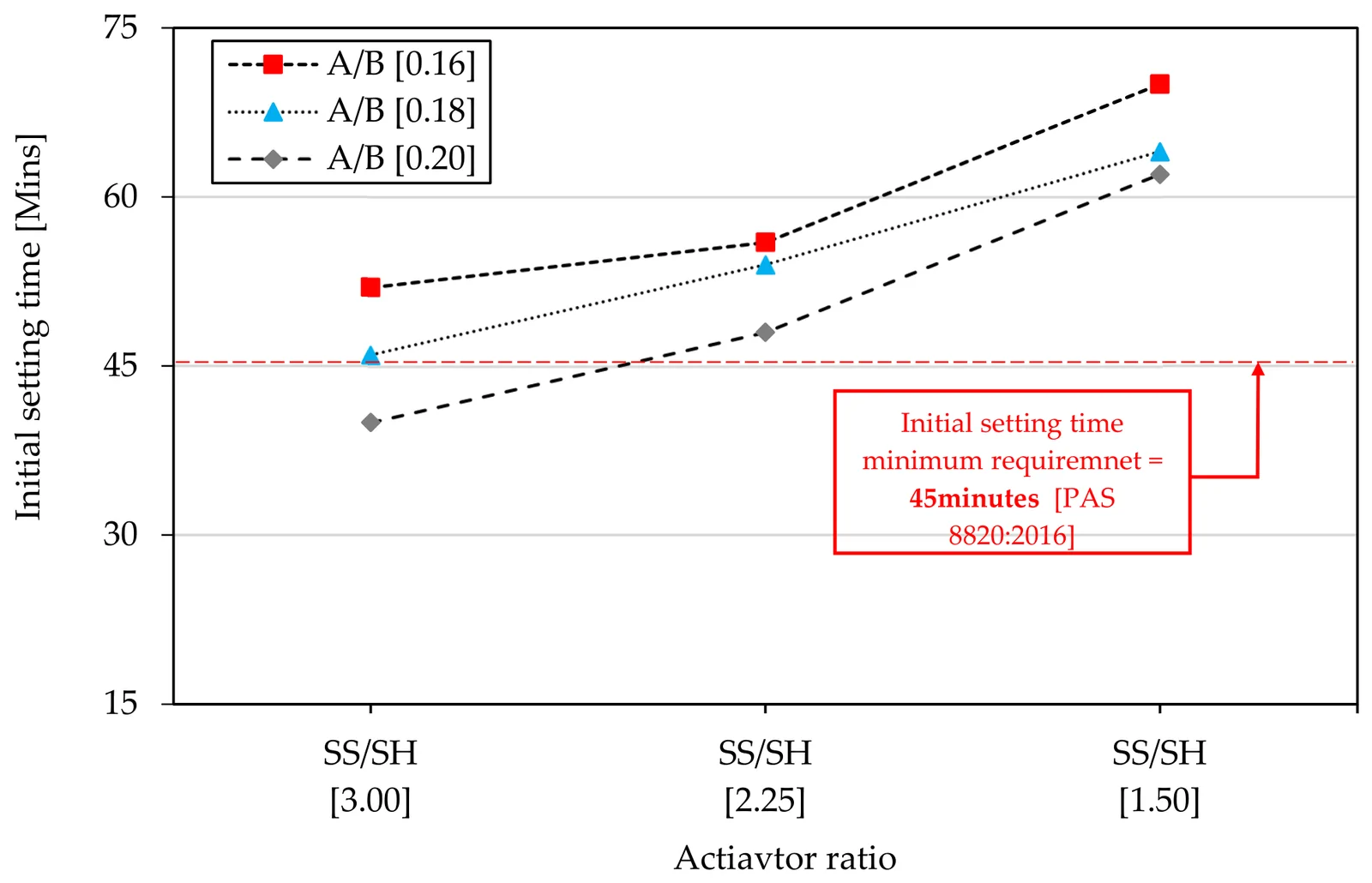
Effect of sodium silicate and sodium hydroxide ratio (SS/SH) for each activator/binder ratio (AB) on initial setting time for Regime B using a 40:60 PFA: GGBS blended AAM and w/b ratio 0.40.

**Figure 6:**
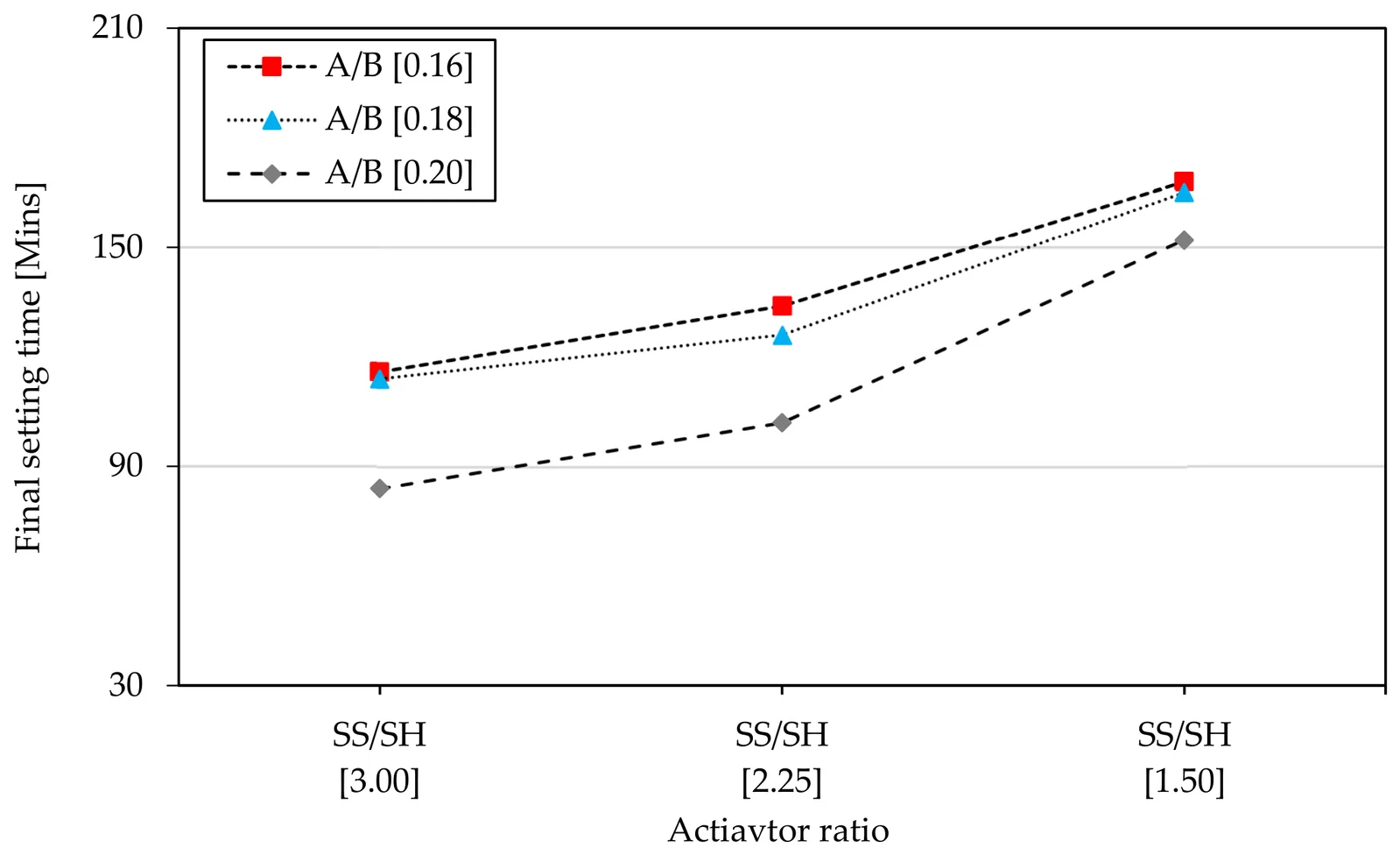
Effect of sodium silicate and sodium hydroxide ratio (SS/SH) for each activator/binder ratio (AB) on final setting time for Regime B using a 40:60 PFA: GGBS blended AAM and w/b ratio 0.40.

Figure 5 and Figure 6 present the initial and final setting time results for the alkali-activated mortar mixes investigated. Except for the mix containing an SS/SH ratio of 3.0 and an A/B ratio of 0.20, all mixes exceeded the minimum setting time threshold of 45 min. The initial and final setting times also exhibited assuring results, with emphasis particularly on gauging the initial setting time, where all, apart from one activator dosage (SS/SH ratio 3.0 and A/B ratio 0.18), surpassed the 45-min minimum threshold in accordance with PAS 8820: 2016. Although the reduction in initial and final setting time caused by increasing the SS:SH from 1.50 to 2.25 and 3.0 directly corresponds with the earlier behavior experienced for workability, attributing the larger volume of soluble silica effectively accelerates the rate of dissolution and reaction kinetics, resulting in a decrease in initial and final setting time [33]. The development in initial and final setting time containing a blend of PFA: GGBS mix composition, chemically activated using commercial SS and SH, has been reported by various research works [9,46], which happen to agree with the test results given in Figure 5 and Figure 6 reporting the setting time is strongly dependent on the SS/SH ratio above 1.5. Conversely, the authors went on to report that no significant change was observed for initial and final setting time using an SS/SH ratio below 1.5.

### 3.2. Comparison Analysis of Alkali-Activated Concrete and Mortar Properties Using Regime A and B

To compare the waste-derived activating solution with the commercial alternative, a suitable activator dosage selected for control purposes was an A/B ratio = 0.18 and an SS/SH ratio of 2.25 combined with a 40:60 PFA: GGBS mix blend proportion and a w/b ratio of 0.40. However, to enable a meaningful comparison between the two systems, the analysis in this section is restricted to SS:SH ratios of 3.00, 2.25, and 1.50.

**Figure 7:**
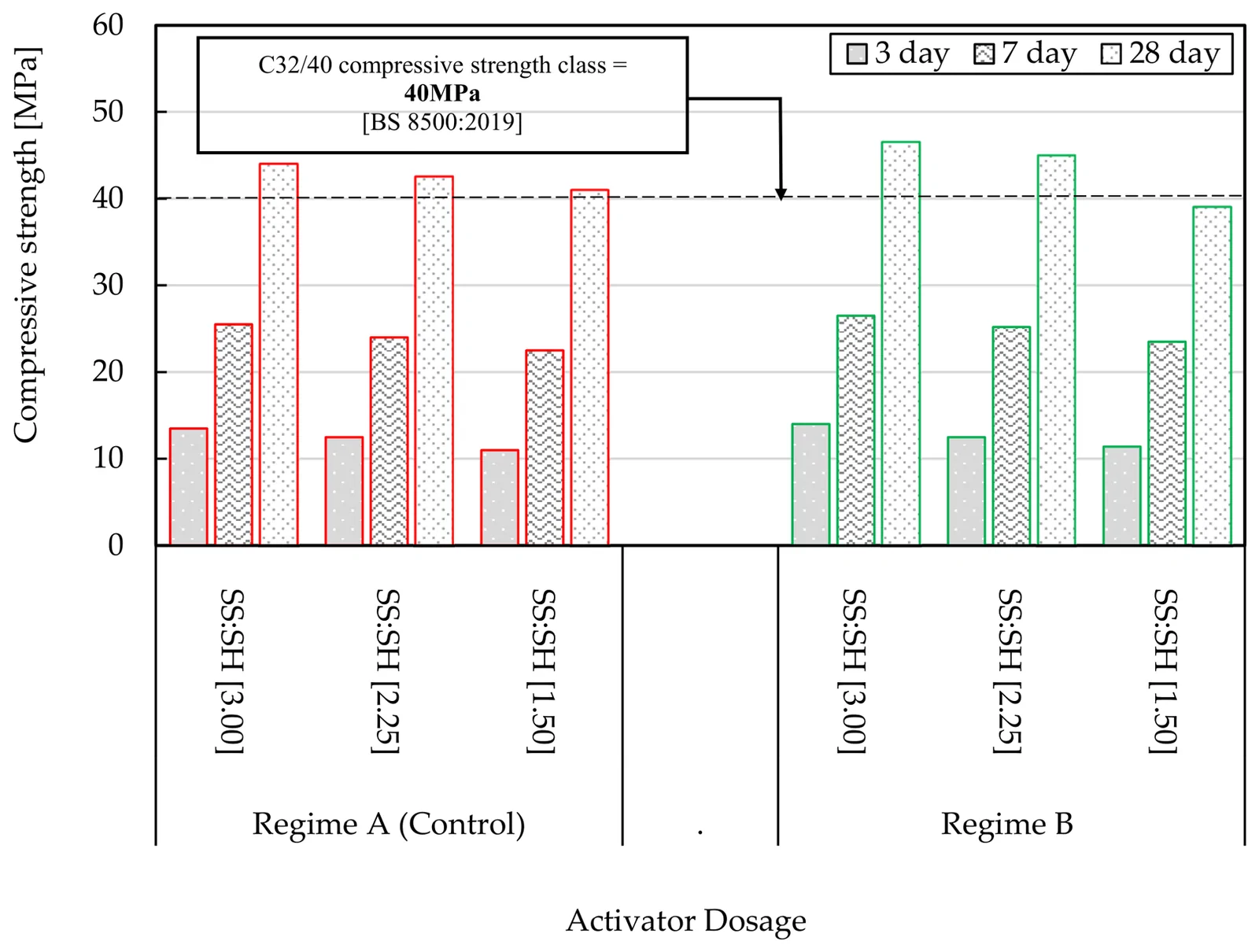
Effect of activator dosage on compressive strength development for a 40:60 PFA: GGBS blended AAC using Regime A and B.

Figure 7 presents the 3-, 7-, and 28-day compressive strength results for the selected mix criteria. Surprisingly, observations show similar CS development results, although Regime B exhibits slightly higher CS development. Furthermore, both regimes exhibit similar trends, with a gradual increase in CS as the SS:SH ratio rises from 1.50 to 3.00.

The initial observation would suggest that the highly amorphous nature of Silica fume was a sensible choice, from a mineralogy perspective, and had a desirable impact during the extraction process. Ref. [50] corroborates this claim, reporting similar findings and stating that the production of a silica waste-derived activator using undensified silica fume exhibited comparable compressive strength to the traditional commercial sodium silicate. Another study published by [4] discusses that the improvement in early strength development using a silica fume-based activator compared with the commercial alternative is dominated by the accelerated rate of available silica species in the alternative activator, which promotes the formation of a highly densified C-(A)-S-H gel structure, consequently enhancing compressive strength. On the contrary, combining with the above theory, there is a possibility that Regime A’s performance is slightly hindered, as the commercial sodium silicate solution in question could potentially have a difference in molar ratio, referring to the SiO_2_ to Na_2_O ratio, compared with that of Regime B, as this could only be provided as a range (1.5 to 2.5) by the supplier. However, the target SiO_2_:Na_2_O ratio adopted during the preparation of the silica fume-derived activator was approximately 2.0, which falls within the specified range provided for the commercial sodium silicate solution. Consequently, whilst minor compositional differences between the activators cannot be entirely excluded, the comparison remains representative of commercially available sodium silicate systems. In general, excessive or a lack of sufficient soluble silica can, however, hinder the polycondensation process, which subsequently has a negative effect on the overall mechanical performance [2,33]. Many researchers have reported on the molar ratio being another key driving parameter for mechanical and microstructural performance [25,51,52]; therefore, it is crucial to determine the actual silica modulus of the activating solutions through further characterization.

Secondly, the comparable strength results suggest exploring alkali concentration (10 M NaOH, earlier recommended by [50], was sufficient for delivering the required alkali ions necessary for extraction purposes, supplying the necessary Si (supplied from silica fume) during the reaction phase. In fact, this desired alkali concentration was an optimal choice for several researchers [4,4,39] for the synthesis of waste-derived silica. The findings directly correspond with [17], which also reported comparable compressive strength results using a silica fume-derived activator from a 10 M NaOH concentration versus commercial sodium silicate.

Lastly, the decision to synthesize the waste-derived activator at room temperature in a 24-h period requiring no additional heat suggests this approach has had a reasonable contribution towards the dissolution of Si derived from silica fume. Nonetheless, the highly amorphous nature of silica fume may have imposed a sufficient amount of activation energy for the dissolution of Si [53] and, therefore, required no unnecessary heating, which could explain the similarities in strength results. Ref. [54] directly agrees with this theory, reporting that silica fume’s high amorphous content is known to have a high value of activation energy, requiring less time and heat during the dissolution process in comparison with other waste sources with lower amorphous content. Ref. [17] experienced comparable compressive strength and setting results using a silica fume-derived activator (versus the commercial alternative) under the same temperature and time constraints. This was not the case for [55], which reported a low solubility of silica derived from RHA using 10 M NaOH solution at room temperature due to a low silica amorphous content, which did not impose sufficient activation energy for the dissolution rate of silica, consequently reducing strength relative to the commercially available alternative.

#### 3.2.1. Workability

**Figure 8:**
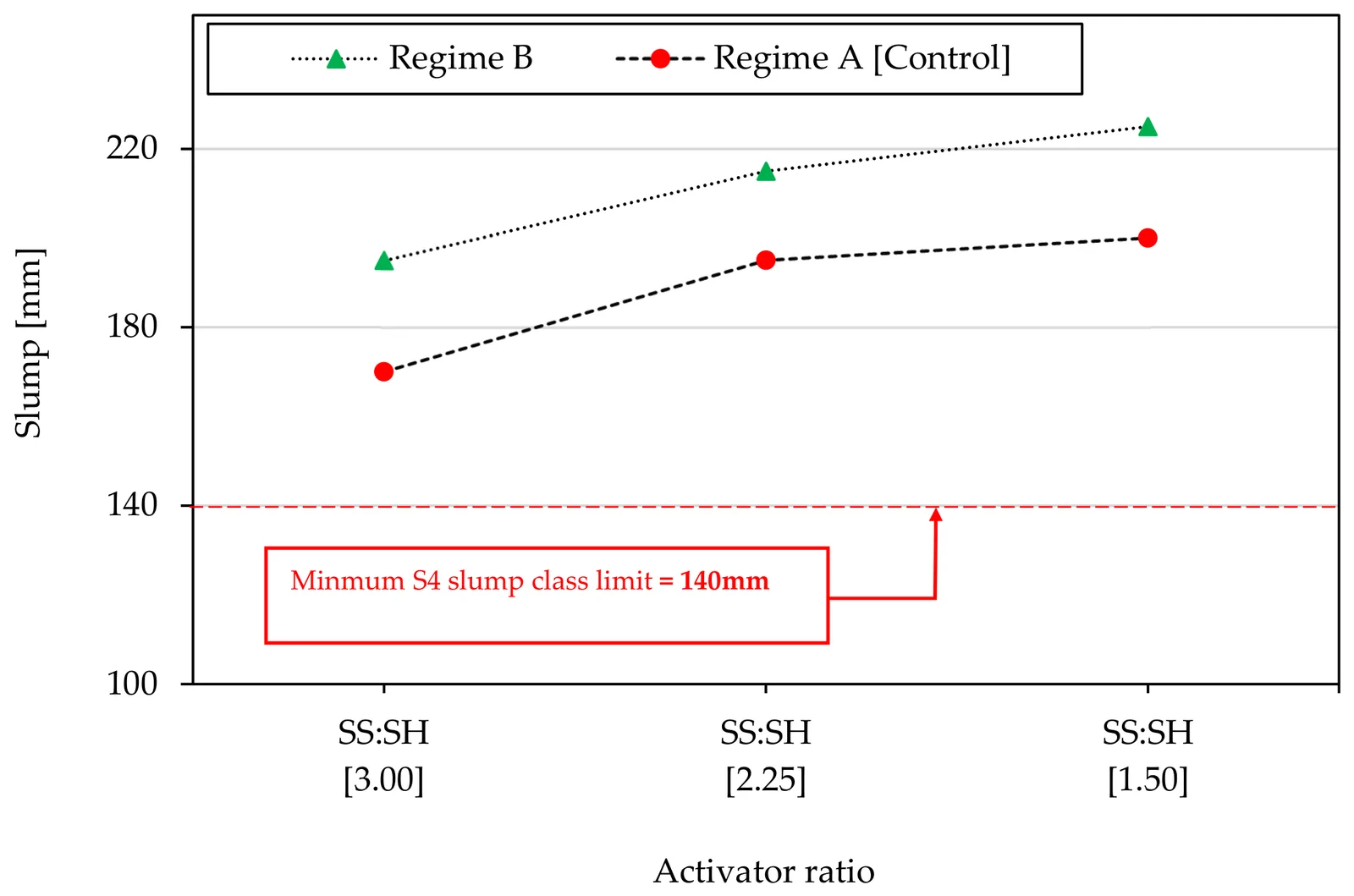
Effect of activator dosage on workability development for a 40:60 PFA: GGBS blended AAC using Regime A and B.

Among the workability test results shown in Figure 8, both Regime A and B greatly exceed the minimum S4 target slump class in accordance with BS EN 206:2013+ A1:2016, whilst presenting a closely similar pattern of behavior when assessing both relationship trends between slump and the respective SS:SH ratios. Yet, unlike the closely similar compressive strength results, a much greater variation in slump is apparent between the two regimes, with Regime B proving to be the far superior performer. Current research evaluating the workability performance of waste-derived activators is scarce. Nonetheless, a key contributing factor that can be attributed to the differences in slump on display can be associated with the silica mineralogy during extraction. When considering the production of commercial sodium silicate solution, there is a broad range of distinctive species of silicate anions, and among them, differences in oligomer molecule sizes influence the reaction kinetics, leading to a significantly viscous solution [56] and subsequently reducing the workability [2]. Given that the exact Si derived during extraction (Regime B) has not been quantified along with viscosity measurements, a strong case can be made that the variation in workability can be attributed to differences in molecule structure and size, which alter the reaction kinetics and viscosity and, fundamentally, overall workability performance. Ref. [57] corroborates this theory, reporting a 30% reduction in consistency (flow table value %) using commercial sodium silicate compared with RHA activator due to increased viscosity, which led to a higher rate of reaction and a reduction in workability.

#### 3.2.2. Setting Time

**Figure 9:**
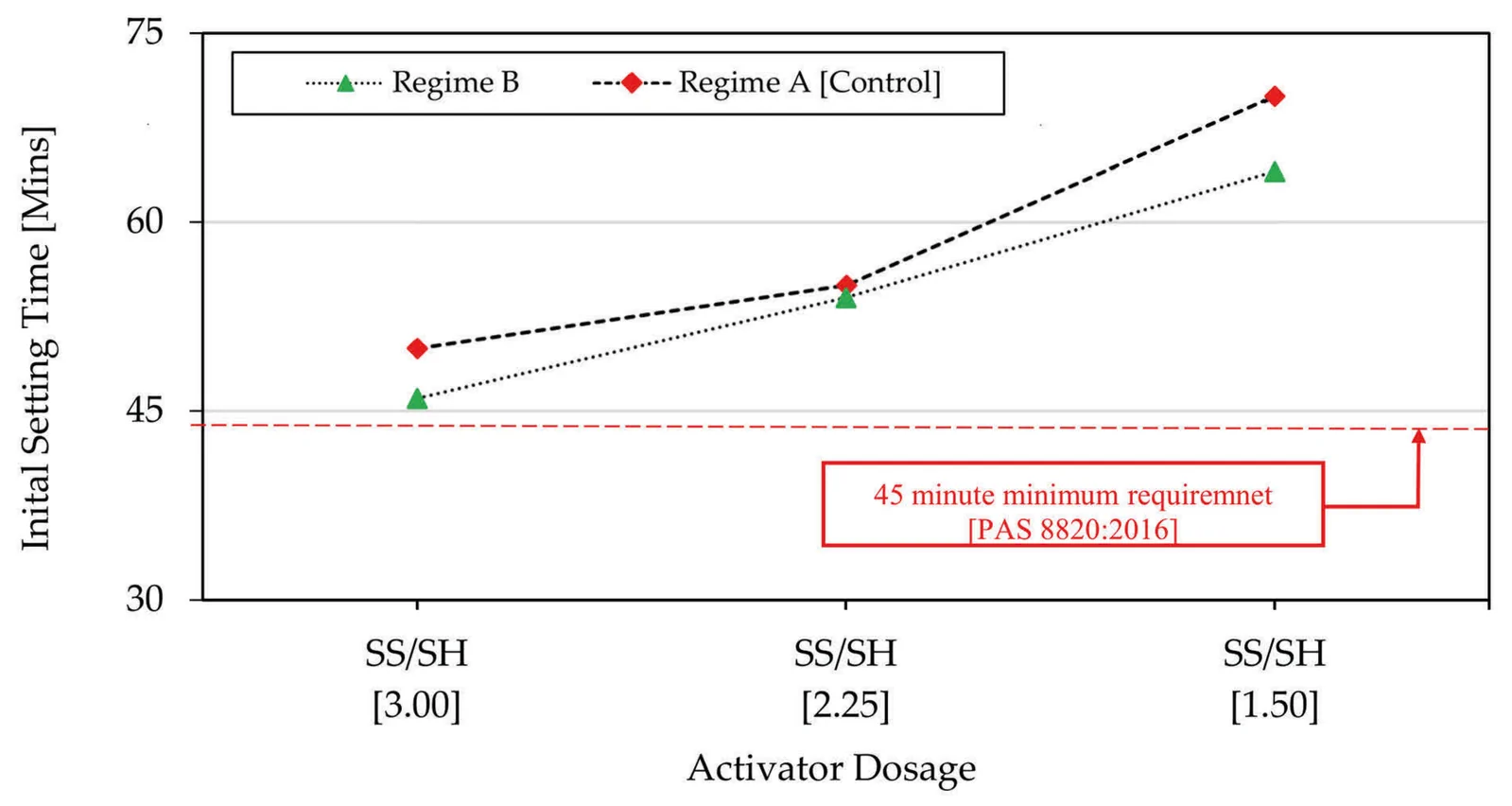
Initial setting time for a 40:60 PFA: GGBS blended AAM using a water-to-binder ratio (0.40), comparing Regime A and B.

**Figure 10:**
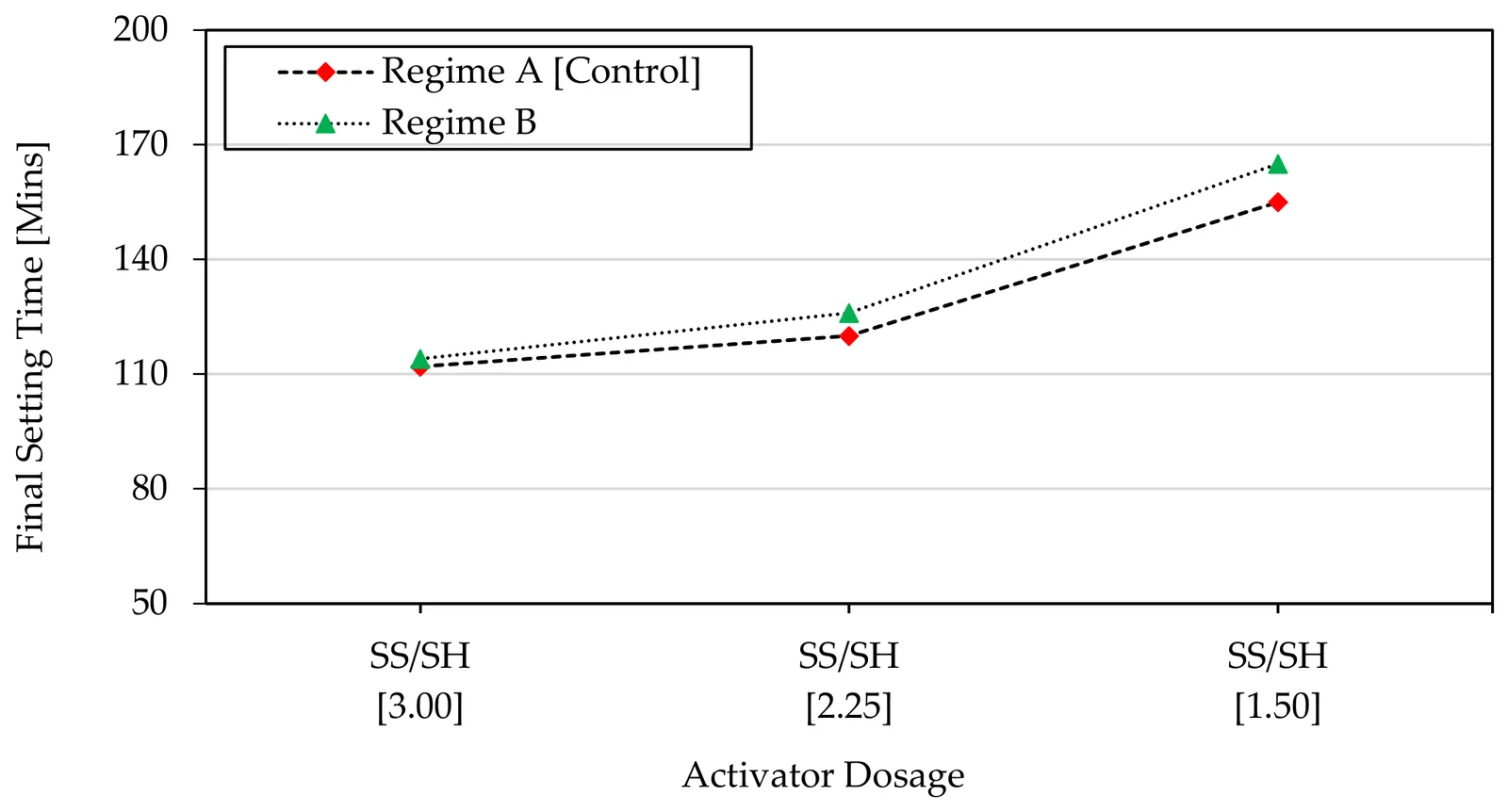
Final setting time for a 40:60 PFA: GGBS blended AAM using a water-to-binder ratio (0.40), comparing Regime A and B.

Closely following the similarities shown for compressive strength, observations shown in Figure 9 and Figure 10 for the initial and final setting time of Regime A and B are very alike, mostly exceeding the minimum 45-min initial requirements in accordance with PAS 8820:2016 The vaguely faster initial setting samples, chemically activated with Regime B directly supports the early assertion made for compressive strength, suggesting the high amorphous nature of silica fume provides a high release rate of silica species during the reaction phase which accelerates the formation of a highly densified gel structure [57], leading to a faster setting material [18]. A study by [8] strengthens this argument, stating that the initial and final setting time of AAM using a rice husk-based activator was reduced due to its high amorphous state versus the commercially available alternative. This was not the case for [54], reporting that the initial and final setting time of AAM using commercial sodium silicate was shorter than that using a rice husk-based activator, with an approximate 19.

Minutes difference in initial setting time. Mejia went on to say this is due to low dissolution of silica with a particularly low amorphous content, which leads to a lower availability of soluble silicate in the solution. This contradiction in the literature confirms that the mineralogical properties, along with quantifying the dissolution of Si, play a vital role in initial and final setting time and therefore would require future investigation to enable accurate quantification. In conjunction with compressive strength, the direct impact of 10 M NaOH concentration, responsible for influencing the extraction of silicate species, can not only lead to higher strength development but also essentially dictates a higher rate of reaction, leading to a faster setting time [5,58]. So it is not surprising that [17] experienced comparable strength and setting time results using the same NaOH concentration (10 M) to synthesize silica fume, compared with commercial sodium silicate (SiO_2_/Na_2_O = 2) [8], which investigated an RHA-derived silicate and commercial silicate solution, along with NaOH used to activated fly ash/slag paste. As a result, the initial and final setting times were largely more rapid using the RHA-derived silicate activator due to a maximum yield of silica (95%) obtained using higher alkali concentrations and a hydrothermal heating process devised to extract soluble silica.

## 4. Conclusions

The findings of this study demonstrate the feasibility of producing geopolymer concrete using a laboratory-synthesized, silica fume-derived sodium silicate solution combined with NaOH as an alternative binary alkali activation system. This approach demonstrates the technical feasibility of producing geopolymer concrete using a laboratory-synthesized silica fume-derived sodium silicate solution combined with NaOH as an alternative binary alkali activation system. Accordingly, the following conclusions are drawn:

1. This study demonstrates that both A/B and SS/SH ratios play a critical role in governing the fresh and hardened properties of alkali-activated systems. Increasing the A/B ratio (0.16–0.20) enhanced compressive strength and reduced setting time, suggesting that this was due to elevated Na^+^ concentrations, which accelerated dissolution and promoted the formation of a dense, homogeneous matrix.
2. Whereas decreasing the A/B ratio reduced the workability properties, suggesting this was attributed to a reduction in liquid, subsequently triggering a larger frictional force between the material particles during mixing.
3. Increasing the SS/SH ratio from 1.50 to 3.0 improved early- and later-age compressive strength while shortening setting times. This behavior may have been attributed to higher soluble silica, which strengthens Si–O–Si bonding, enhances reaction kinetics, and supports the development of a more uniform microstructure.
4. Enhancing the SS/SH ratio from 1.50 to 3.0 reduced the initial and final slump setting times, indicating this could have been attributed to an accelerated rate of dissolution and reaction kinetics, resulting in a faster initial and final setting time. The rise in SS content was also claimed to have enhanced viscosity, potentially leading to stickier material, which could explain the gradual reduction in slump.
5. Similar fresh and hardened performance was observed for the commercial (Regime A) and waste-derived activator (Regime B) within the scope of the experimental program, highlighting the effectiveness of silica fume as a precursor when chemically activated with sodium hydroxide. The observed behavior may be associated with the high amorphous silica content of the silica fume, which has been reported to enhance dissolution and reaction kinetics in similar alkali-activated systems. However, as direct characterization of the reaction products was not undertaken in the present study, this interpretation should be regarded as a literature-supported theory, rather than direct evidence of a specific reaction mechanism. Further microstructural investigation is required to verify the underlying reaction mechanisms and reaction products.
6. 10 M NaOH concentration was sufficient to achieve effective silica dissolution. Importantly, room-temperature extraction over 24 h achieved comparable engineering performance without the need for additional thermal treatment, demonstrating the feasibility of producing a silica fume-derived activator under ambient conditions. However, the environmental implications associated with this preparation method were not quantified within the scope of the present study.
7. Minor differences in workability between Regime A and B are likely linked to variations in silicate species, molecular structure, and viscosity. While commercial solutions contain diverse silicate oligomers, the chemistry of the extracted activator remains insufficiently quantified.
8. While the laboratory-developed silica fume–derived activated solution proved effective, its conversion to field application requires careful consideration. Consistent material performance in large-scale production depends on controlled storage conditions for both precursors and activators, alongside the use of mechanical or ready-mix batching processes to replicate laboratory mixing conditions.
9. Although the present study demonstrates the engineering feasibility of the silica fume-derived activator, the underlying reaction mechanisms and phase development were not investigated. Future work should incorporate microstructural and mineralogical characterization techniques, including scanning electron microscopy (SEM), X-ray diffraction (XRD), and Fourier-transform infrared spectroscopy (FTIR), to provide a more detailed understanding of the reaction products and their relationship to the observed engineering performance.
10. Whilst workability and setting behavior were evaluated in the present study, detailed rheological characterization was beyond the scope of the experimental program. Future research should investigate rheological parameters such as yield stress, plastic viscosity, and thixotropic behavior to improve understanding of the influence of silica fume-derived activators on the fresh-state performance of alkali-activated concrete.

The results demonstrate that a silica fume-derived sodium silicate activator produced under ambient-temperature conditions can achieve engineering performance comparable to that of a commercial sodium silicate activator. Whilst previous studies have suggested that waste-derived activators may offer environmental advantages through the utilization of industrial by-products and reduced reliance on conventionally manufactured activators, the environmental performance of the proposed activator was not quantified within the scope of this study. Consequently, dedicated life-cycle, embodied carbon, and techno-economic assessments are recommended as part of future research.

## Figures and Tables

**Table 1:** Summaryof previous studies and positioning of the present research.

Study	Focus	Key Contribution
[17]	Proof-of-concept	Demonstrated synthesis of silica fume-derived sodium silicate at paste scale.
[15]	Concrete performance	Demonstrated the viability of silica fume-derived sodium silicate in GGBS-based geopolymer concrete and assessed fresh (workability only) and hardened engineering properties.
[23]	Activator preparation	Investigated activator dissolution time and mixing methodology.
[24]	Precursor optimization	Investigated optimum PFA-GGBS precursor combinations.
Present Study	Activator optimization	Systematic evaluation of SS/SH and A/B ratios within a PFA-GGBS system, including setting behavior assessment and benchmarking against a commercial sodium silicate activator to establish practical dosage guidance and comparison analysis.

**Table 4:** The mechanical and physical properties of the aggregates used in this research.

Properties	Fine Aggregate	Coarse Aggregates	
	Sand	10/4 mm	20/10 mm
Water Absorption (%)	0.85	1.6	1.2
Saturated Density (kg/m^3^)	2820	2620	2580
Dry Density (kg/m^3^)	2710	2580	2540

**Table 5:** Chemical and physical properties of alkali-activators.

Properties	Sodium Hydroxide	Sodium Silicate	(SF) NaOH-Based Sodium Silicate
SiO_2_:Na_2_O ratio	-	1.5–2.5	2
Density (kg/m^3^)	1310	1560	≈1560
pH	-	11.2	-

**Table 8:** Comparison analysis of AAC mix design for 1 m^3^ for Regime A and B with respect to SS/SH ratios 3:1, 2.25:1, and 1.50:1 and (AB) ratio 0.18.

Mix Code	Alkali-Activated Binder				Water (kg/m^3^)	Aggregates (kg/m^3^)		
	GGBS (kg/m^3^)	PFA (kg/m^3^)	A/B Ratio	SS:SH Ratio		Sand	10 mm	20 mm
RA63	225.4	150.3	0.18	3.00	149.5	751.2	450.72	676.08
RA62	225.4	150.3	0.18	2.25	149.5	751.2	450.72	676.08
RA61	225.4	150.3	0.18	1.50	149.5	751.2	450.72	676.08
RB73	225.4	150.3	0.18	3.00	149.5	751.2	450.72	676.08
RB72	225.4	150.3	0.18	2.25	149.5	751.2	450.72	676.08
RB71	225.4	150.3	0.18	1.50	149.5	751.2	450.72	676.08

## Data Availability

The original contributions presented in this study are included in the article. Further inquiries can be directed to the corresponding authors.

## References

[1] Andres R.J., Boden T.A., Bréon F.-M., Ciais P., Davis S., Erickson D., Gregg J.S., Jacobson A., Marland G., Miller J. (2012). A synthesis of carbon dioxide emissions from fossil-fuel combustion. Biogeosciences.

[2] Rodríguez E.D., Bernal S.A., Provis J.L., Paya J., Monzo J.M., Borrachero M.V. (2013). Effect of nanosilica-based activators on the performance of an alkali-activated fly ash binder. Cem. Concr. Compos..

[3] Scrivener K.L., John V.M., Gartner E.M. (2018). Eco-efficient cements: Potential economically viable solutions for a low-CO2 cement-based materials industry. Cem. Concr. Res..

[4] Bernal S., Rodríguez E., Mejía De Gutiérrez R., Provis J.L. (2015). Performance at high temperature of alkali-activated slag pastes produced with silica fume and rice husk ash based activators. Mater. Constr..

[5] Phoo-Ngernkham T., Phiangphimai C., Intarabut D., Hanjitsuwan S., Damrongwiriyanupap N., Li L.-Y., Chindaprasirt P. (2020). Low cost and sustainable repair material made from alkali-activated high-calcium fly ash with calcium carbide residue. Constr. Build. Mater..

[6] Lyu B.-C., Ding C., Guo L.-P., Chen B., Wang A.-G. (2021). Basic performances and potential research problems of strain hardening geopolymer composites: A critical review. Constr. Build. Mater..

[7] Rafeet A., Vinai R., Soutsos M., Sha W. (2019). Effects of slag substitution on physical and mechanical properties of fly ash-based alkali activated binders (AABs). Cem. Concr. Res..

[8] Tong K.T., Vinai R., Soutsos M.N. (2018). Use of Vietnamese rice husk ash for the production of sodium silicate as the activator for alkali-activated binders. J. Clean. Prod..

[9] Topark-Ngarm P., Chindaprasirt P., Sata V. (2015). Setting time, strength, and bond of high-calcium fly ash geopolymer concrete. J. Mater. Civ. Eng..

[10] Wang A., Zheng Y., Zhang Z., Liu K., Li Y., Shi L., Sun D. (2020). The Durability of Alkali-Activated Materials in Comparison with Ordinary Portland Cements and Concretes: A Review. Engineering.

[11] Xie T., Visintin P. (2018). A unified approach for mix design of concrete containing supplementary cementitious materials based on reactivity moduli. J. Clean. Prod..

[12] Zhang P., Gao Z., Wang J., Guo J., Hu S., Ling Y. (2020). Properties of fresh and hardened fly ash/slag based geopolymer concrete: A review. J. Clean. Prod..

[13] Provis J.L., Palomo A., Shi C. (2015). Advances in understanding alkali-activated materials. Cem. Concr. Res..

[14] Witherspoon R., Wang H., Aravinthan T., Omar T. (2009). Energy and emissions analysis of fly ash based geopolymers. SSEE 2009: Solutions for a Sustainable Planet.

[15] Adeleke B.O., Kinuthia J.M., Oti J., Ebailila M. (2023). Physico-Mechanical Evaluation of Geopolymer Concrete Activated by Sodium Hydroxide and Silica Fume-Synthesised Sodium Silicate Solution. Materials.

[16] Alnahhal M.F., Kim T., Hajimohammadi A. (2021). Waste-derived activators for alkali-activated materials: A review. Cem. Concr. Compos..

[17] Billong N., Oti J., Kinuthia J. (2021). Using silica fume based activator in sustainable geopolymer binder for building application. Constr. Build. Mater..

[18] Kumar S.S., Rithuparna R., Senthilkumar R., Bahurudeen A. (2023). Cleaner production of waste-derived alkali activators from industrial and agricultural by-products for sustainable alkali activated binders. Constr. Build. Mater..

[19] Luga E., Atiş C.D. (2016). Strength Properties of Slag/Fly Ash Blends Activated with Sodium Metasilicate and Sodium Hydroxide+ Silica Fume. Period. Polytech. Civ. Eng..

[20] Ziegler D., Formia A., Tulliani J.M., Palmero P. (2016). Environmentally-Friendly Dense and Porous Geopolymers Using Fly Ash and Rice Husk Ash as Raw Materials. Materials.

[21] Samarakoon M.H., Ranjith P.G., Wanniarachchi W.A.M. (2021). Properties of well cement following carbonated brine exposure under HTHP conditions: A comparative study of alkali-activated and class G cements. Cem. Concr. Compos..

[22] Wu Y., Jia Z., Qi X., Wang W., Guo S. (2024). Alkali-activated materials without commercial activators: A review. J. Mater. Sci..

[23] Altameemi S., Casabuena L.R., Oti J., Kinuthia J.M. (2025). Understanding the Effect of Waiting for the Dissolution of Sodium Hydroxide in Geopolymer Concrete Mixes. Materials.

[24] Oti J., Adeleke B.O., Casabuena L.R., Kinuthia J.M., Sule S. (2025). Utilization of a PFA-GGBS-Based Precursor in Geopolymer Concrete Production as a Sustainable Substitute for Conventional Concrete. Materials.

[25] (2012). Fly Ash for Concrete: Definition, Specifications and Conformity Criteria.

[26] (2006). Ground Granulated Blast Furnace Slag for Use in Concrete, Mortar and Grout: Definitions, Specifications and Conformity Criteria.

[27] (2010). Silica Fume for Concrete: Conformity Evaluation.

[28] (2013). Aggregates for Concrete.

[29] (2020). Concrete. Complementary British Standard to BS EN 206: Method of Specifying and Guidance for the Specifier.

[30] (2019). Testing Fresh Concrete: Slump Test.

[31] (2016). Construction Materials. Alkali-Activated Cementitious Material and Concrete. Specification.

[32] Rafeet A.M.I. (2016). Mix Design, Fresh and Hardened Properties and Microstructural Characterisation of Alkali-Activated Concrete Based on PFA/GGBS Blends. Ph.D. Thesis.

[33] Xie T., Visintin P., Zhao X., Gravina R. (2020). Mix design and mechanical properties of geopolymer and alkali activated concrete: Review of the state-of-the-art and the development of a new unified approach. Constr. Build. Mater..

[34] Jiao Z., Wang Y., Zheng W., Huang W. (2018). Effect of Dosage of Alkaline Activator on the Properties of Alkali-Activated Slag Pastes. Adv. Mater. Sci. Eng..

[35] Kamseu E., Beleuk à Moungam L.M., Cannio M., Billong N., Chaysuwan D., Melo U.C., Leonelli C. (2017). Substitution of sodium silicate with rice husk ash-NaOH solution in metakaolin based geopolymer cement concerning reduction in global warming. J. Clean. Prod..

[36] (2006). Admixtures for Concrete, Mortar and Grout. Test Methods. Part 2: Determination of Setting Time.

[37] (2016). Methods of Testing Cement. Part 3: Determination of Setting Times and Soundness.

[38] (2011). Testing Hardened Concrete: Compressive Strength of Test Specimens.

[39] Bernal S.A., Rodríguez E.D., Mejia De Gutiérrez R., Provis J.L., Delvasto S. (2011). Activation of Metakaolin/Slag Blends Using Alkaline Solutions Based on Chemically Modified Silica Fume and Rice Husk Ash. Waste Biomass Valorization.

[40] Sindhunata, Van Deventer J., Lukey G., Xu H. (2006). Effect of curing temperature and silicate concentration on fly-ash-based geopolymerization. Ind. Eng. Chem. Res..

[41] Sun B., Ye G., De Schutter G. (2022). A review: Reaction mechanism and strength of slag and fly ash-based alkali-activated materials. Constr. Build. Mater..

[42] Hardjito D., Wallah S.E., Sumajouw D.M., Rangan B.V. (2004). On the development of fly ash-based geopolymer concrete. Mater. J..

[43] Joseph B., Mathew G. (2012). Influence of aggregate content on the behavior of fly ash based geopolymer concrete. Sci. Iran..

[44] Provis J.L. (2018). Alkali-activated materials. Cem. Concr. Res..

[45] Nath P., Sarker P.K. (2014). Effect of GGBFS on setting, workability and early strength properties of fly ash geopolymer concrete cured in ambient condition. Constr. Build. Mater..

[46] Fang G., Ho W.K., Tu W., Zhang M. (2018). Workability and mechanical properties of alkali-activated fly ash-slag concrete cured at ambient temperature. Constr. Build. Mater..

[47] Zhang P., Wang K., Li Q., Wang J., Ling Y. (2020). Fabrication and Engineering Properties of Concretes Based on Geopolymers/Alkali-activated Binders-A Review. J. Clean. Prod..

[48] Yaseri S., Hajiaghaei G., Mohammadi F., Mahdikhani M., Farokhzad R. (2017). The role of synthesis parameters on the workability, setting and strength properties of binary binder based geopolymer paste. Constr. Build. Mater..

[49] Sathonsaowaphak A., Chindaprasirt P., Pimraksa K. (2009). Workability and strength of lignite bottom ash geopolymer mortar. J. Hazard. Mater..

[50] Billong N., Kinuthia J., Oti J., Melo U.C. (2018). Performance of sodium silicate free geopolymers from metakaolin (MK) and Rice Husk Ash (RHA): Effect on tensile strength and microstructure. Constr. Build. Mater..

[51] Adam A. (2009). Strength and Durability Properties of Alkali Activated Slag and Fly Ash-Based Geopolymer Concrete. Ph.D. Thesis.

[52] Marjanović N., Komljenović M., Baščarević Z., Nikolić V., Petrović R. (2015). Physical–mechanical and microstructural properties of alkali-activated fly ash–blast furnace slag blends. Ceram. Int..

[53] Garcia Lodeiro I., Cristelo N., Palomo A., Fernández-Jiménez A. (2020). Use of industrial by-products as alkaline cement activators. Constr. Build. Mater..

[54] Mejía J.M., Mejía De Gutiérrez R., Montes C. (2016). Rice husk ash and spent diatomaceous earth as a source of silica to fabricate a geopolymeric binary binder. J. Clean. Prod..

[55] Luukkonen T., Abdollahnejad Z., Yliniemi J., Kinnunen P., Illikainen M. (2018). Comparison of alkali and silica sources in one-part alkali-activated blast furnace slag mortar. J. Clean. Prod..

[56] Bahlmann E.F., Harris R., Rockliffe J., Smith E. (1997). Silicon-29 NMR self-diffusion and chemical-exchange studies of concentrated sodium silicate solutions. J. Chem. Soc. Faraday Trans..

[57] Das S.K., Behera N., Patro S.K., Mustakim S.M., Suda Y., Leklou N. (2023). Effectiveness of rice husk ash-derived alkali activator in fresh, mechanical, and microstructure properties of geopolymer mortar at ambient temperature curing. J. Sustain. Cem.-Based Mater..

[58] Perná I., Hanzlíček T. (2016). The setting time of a clay-slag geopolymer matrix: The influence of blast-furnace-slag addition and the mixing method. J. Clean. Prod..

[59] Ayres A. (2025). The Performance of a Blended PFA/GGBS Alkali-Activated Concrete Using a Silica Fume-Based Activator. Ph.D. Thesis.

